# How can task shifting put patient safety at risk? A qualitative study of experiences among general practitioners in Norway

**DOI:** 10.1080/02813432.2020.1714143

**Published:** 2020-01-23

**Authors:** Kirsti Malterud, Aase Aamland, Anette Fosse

**Affiliations:** a Research Unit for General Practice, NORCE Norwegian Research Centre, Bergen, Norway;; b Department of Global Public Health and Primary Care, University of Bergen, Bergen, Norway;; c The Research Unit and Section of General Practice, Department of Public Health, University of Copenhagen, Copenhagen, Denmark;; d Health, Care and Social Services, County Governor of Agder, Arendal, Norway;; e National Centre of Rural Medicine, Department of Community Medicine, Faculty of Health Sciences, University of Tromsø, Tromsø, Norway

**Keywords:** General practice, task shifting, patient safety, adverse events, qualitative method

## Abstract

**Objective:** To describe experiences among general practitioners (GPs) in Norway regarding horizontal task shifting experiences associated with adverse events that potentially put patient safety at risk.

**Design and contributors:** We conducted a qualitative study with data from a retrospective convenience sample of consecutive, already posted comments in a restricted Facebook group for GPs in Norway. The sample consisted of 43 unique posts from 38 contributors (23 women and 15 men), presenting thick and specific accounts of potentially adverse events in the context of horizontal task shifting. Analysis was conducted with systematic text condensation, a method for thematic cross-case analysis.

**Results:** Contributing GPs reported several types of adverse events associated with horizontal task shifting that could put patient safety at risk. They described how spill-over work dispatched to GPs may generate administrative hassle and hazardous delay of necessary examinations. Overdiagnosis, reduced access and endangered accountability occur when time-consuming procedures and pre-investigation before referral are pushed upon GPs. Resource-draining chores beyond GPs’ proficiency is also dispatched without appropriate instruction or equipment. Furthermore, potential malpractice is imposed by hospital colleagues who overrule the GPs’ medical judgement.

**Implications:** Patient safety is endangered when horizontal task shifting is initiated and performed without a systematic process involving all stakeholders that considers available resources. A risk and vulnerability analysis, securing competent staff, resources, time and equipment before launching such reforms is necessary to protect patient safety. Infrastructure comprised of local coordination groups may facilitate dialogue between health care service levels and negotiate responsibilities and workload.

**Key points**
- Task shifting between different levels of health care is a relevant and legitimate strategy for planning and policy.
- GPs in Norway report adverse events related to task shifting from specialist colleagues without proper resource allocation.
- Patient safety may be put at risk by hazardous delay, overdiagnosis, endangered accountability and potential malpractice.
- Planning and implementation of task shifting must involve all system levels and relevant stakeholders to ensure patient safety.

## Introduction

Task shifting between different health care levels is a relevant, legitimate strategy for planning and policy, and it is usually motivated by best possible utilization of limited resources [1–3]. Task shifting is usually vertically staged, with tasks formally transferred from a higher level of competence to a lower one, such as from doctors to nurses or from nurses to lay persons [3]. Horizontal substitution occurs when tasks are shifted between levels of equivalent professional competence [4], such as from hospital specialists and other specialist colleagues to GP specialists. Since 2012, substantial horizontal task shifting has taken place in Norway in the wake of the Coordination Reform (CR). This reform was launched to improve patient trajectories and provide cost-effective services closer to patients [5,6]. Driven by economic and legal incentives, all municipalities were for example instructed to establish institutions with emergency care beds run by GPs [7]. The aim was to avoid unnecessary hospital admissions.

With this reform, GPs also experienced a staggering increase in their responsibilities to enlisted patients, reported by supporters as well as resisters of the reform itself. A propensity towards task shifting subsequent to the CR was found to affect the GPs’ time and resources and to substantially contribute to the current Norwegian GP recruitment crisis [8,9]. Media reported examples of both formal tasks shifting based on different national guidelines and procedures and informal task shifting evolving without underlying evidence-based assessments, official decisions or corresponding resources.

Often, such reforms are soundly based and well-prepared with adequate organizational assets, but concerns have also been raised regarding whether task shifting leads to inferior services for vulnerable groups [10]. Patient safety is the prevention of errors and adverse effects to patients associated with health care [11]. According to Safety Improvement for Patients in Europe (SimPatIE), adverse events are situations involving a potential risk of patient harm, without harm having necessarily occurred [12]. Having served as GPs ourselves for several decades in urban and rural settings, our preconception was that task shifting, especially horizontal and informal, could lead to major hassle for patients’ rights and GPs’ operational conditions as well as adverse events threatening patient safety. We had experienced how apparently minor negative impacts may sometimes hold the potential for more serious consequences. When task shifting is considered a strategy for organizational change in health care, the impact on patient safety must be assessed. Therefore, we conducted a study among GPs in Norway describing horizontal task shifting experiences associated with adverse events and major hassles that potentially put patient safety at risk.

## Design, material and methods

We conducted a qualitative study analyzing texts reporting GPs’ horizontal task shifting experiences. Data were drawn from a retrospective convenience sample of previously posted comments in a restricted Facebook group for GPs in Norway. When data collection was initiated, the list (Allmennlegeinitiativet – the GP Initiative) included 3840 of the approximately 4800 GPs working in Norway, 2784 of whom were active participants [13]. We collected posted data within two months during fall 2018, a period when vivid discussions about task shifting took place. Often, these posts lead to further discussion with threads of contributions. To maintain contextual information, we preferred posts initiating a thread. Only contributions where we interpreted horizontal task shifting to imply adverse events potentially endangering patient safety were included. We emphasized examples of informal task shifting, but the border towards formal task shifting was not always clear. The sample consisted of 43 unique posts from 38 contributors (23 women and 15 men). Eighteen contributors stated their age (range 27–64 years, median 40 years). Most presented thick, specific descriptions [14] of varied relevant events. We assessed this corpus of texts (range 14–915 words, each typically around 200 words) to ascertain appropriate information power [15] for analysis.

Analysis was conducted with systematic text condensation [16], a method for thematic cross-case analysis of qualitative data, commonly applied in medical qualitative research in Scandinavia due to its transparency and methodical approach. Analysis was conducted according to the following steps: (a) read the material to gain an overview and elicit preliminary themes, (ii) develop code groups from preliminary themes, then identify and sort meaning units reflecting the contributors’ experiences of task shifting associated with adverse events, (iii) establish subgroups exemplifying vital aspects of every code group by condensing the contents of each and identifying illustrating quotes and (iv) synthesize the condensates from each code group to reconceptualized descriptions of adverse events that indicate potentially hazardous horizonal task shifting. KM and AF conducted the main part of analysis by negotiating code groups and subgroups. Analysis was further elaborated on by AA in the later steps of writing. Perspectives and concepts about patient safety [12] and dynamic professional boundaries in the health care workforce [4] supported our analysis, though not as a template framework [17].

## Research ethics

The study was carried out in accordance with The Code of Ethics of the World Medical Association with informed consent obtained retrospectively. None of the enquired contributors refused to participate. The Facebook group enforces strict rules on confidentiality, with any case report properly anonymized. The moderator accepted and supported the study. The Regional Committee for Ethics in Medical Research assessed the study and concluded it was outside of their mandate (2019/35/REK vest). The Norwegian Social Science Data Services (NSD) approved the study (# 938295/2019).

## Results

Contributing GPs reported several types of adverse events associated with horizontal task shifting that could put patient safety at risk. They described how spill-over work dispatched to GPs may generate administrative hassle and hazardous delay of necessary examinations. Overdiagnosis, reduced access and endangered accountability occur when time-consuming procedures and pre-investigation before referral are pushed upon GPs. Resource-draining chores beyond GPs’ proficiency is also dispatched without appropriate instruction or equipment. Furthermore, potential malpractice is imposed by hospital colleagues who overrule the GPs’ medical judgement. The respective contributors have been assigned pseudonyms.

### Spill-over work dispatched to GPs can generate administrative hassle and hazardous delay

Several contributors described situations where administrative tasks inappropriately dispatched to the GP led to harmful or potentially hazardous delay and subsequent patient annoyance. Some described hospitalized patients who were asked to contact their GP for a sick leave certificate, which according to law should be issued by the doctor in charge. If the GP refused such a request, the patient would be left in a bad situation. Other examples concerned medical interaction regarding the license to drive. Some contributors mentioned patients who had been hospitalized for fainting without any serious illness being diagnosed. The hospital doctor had not drawn any conclusions, but patients were still temporarily forbidden to drive. When these patients complained, they were told to contact their GP, even though the hospital doctor was responsible and held the relevant information. One frustrated GP said:

"One of my patients got a verbal driving ban from the hospital specialist “just in case” after hospital admission due to a probable vasovagal syncope. This driving ban had, however, enormous economic consequences for a healthy transport driver and his business. He complained to the hospital, but was met with a verbal message that his GP had to sort this out… "(Ann)

A common experience among the contributors was that the specialists to whom the patients had been referred wanted further examinations (such as MRI or colonoscopy) to be conducted. Instead of transferring the patients further, they issued a request that the GPs do this, even demanding the GPs to convey the reports back to the hospital. Patients examined by specialists or private medical services had presented alarming symptoms and findings, but follow-up was left to the patients themselves or to the GP. One patient injured his arm and contacted a private internet doctor service where MRI was ordered. The scan disclosed tendon injury, but no treatment or follow-up was offered by the internet doctor and the GP received no information until later. Another patient had been referred by his GP to the hospital due to cancer suspicion. He underwent a rectoscopy, which was normal. The patient was then told to contact his GP for a re-referral to colonoscopy. One GP received a comprehensive summary of the patient’s story from a hospital specialist, who demanded that the GP quickly should refer the patient for further examinations. Another contributor in a similar situation stated the following:

"After having written a polite answer to the specialist about how irrational I thought this solution was, I received today a phone call from this colleague who told me that she totally agreed and appreciated my response. I am content that I finally took my time to do this!" (Roger)

### Overdiagnosis, reduced access and endangered accountability may occur when time-consuming procedures and pre-investigations are inflicted on GPs

Contributors described examples of horizontal task shifting stemming from comprehensive procedures triggered by guidelines from health authorities and hospital departments. Typically, such demands assigned the GPs many inappropriate responsibilities that required a wide range of physical examinations and screening tests before the hospital gate could be opened. These tasks would not only create a possible delay for the actual patients, they would also induce overdiagnosis and divert time that could have been allocated to other patients.

Two contributors mentioned a recently launched care pathway for patients with psychiatric symptoms or drug abuse problems, acknowledging GPs’ important functions within this field. However, their accounts described how GPs are expected to know the full details of all requested procedures, handle the referral recommendations, offer comprehensive investigation of the patient’s physical health and collaborate with specialists, municipal health services and user organizations. Another example dealt with tasks initiated by child protection services. Even with the independent referral mandate, the child protection services still expected GPs to provide supplementary health examinations and information according to guidelines developed and implemented with little or no GP input. In one municipality, the 20 GPs were not even invited when the child and adolescent psychiatric service arranged an information meeting. Other contributors mentioned extensive investigations for children adopted from abroad, where the GP was instructed to conduct clinical examinations, screen for hereditary diseases and MRSA, conduct psychosocial investigations, administer vaccines, review dental development and conduct fecal tests. A contributor expressed worries about accountability:

"In principle, these systems impose the legal responsibility of consequences of delay or impediment in the chain of referral, since the referral can be denied if it is not sufficiently complete. I guess this is a risk of ending up in a cleft stick." (Billy)

The contributors described how referrals could trigger a response from the addressee, ordering the GP to conduct a comprehensive pre-investigation program before any assessment would be initiated. Two of them described detailed lists of orders—up to 10 pages—received from hospital departments when they had referred patients for a potential attention deficit hyperactivity disorder diagnosis. These lists deviated from national recommendations, especially regarding who was responsible for the diagnostic investigations. The GPs were assigned responsibility for all these orders, with one of the lists specifying more than 100 items to be checked before the child and youth mental health specialist would see the patient. One of the GPs commented that many of these orders, including physical examinations, would have been more appropriately and specifically conducted in the hospital. Similar experiences were reported from referrals to a drug abuse program, where the clinic responded with comprehensive pre-assessment requirements. A contributor discussed these wide-ranging requests as follows:

"I had a letter from the mental health clinic about pre-investigation of patients for whom ECT treatment was being planned. They asked the GP to take a lot of blood tests, conduct a full physical and neurological status including ophthalmoscopy, ECG, refer to computer tomography of the head and chest X-ray. How are such questions managed in other places? I guess the psychiatrist at the mental health clinic could himself better do a lot of these things?" (Elizabeth)

### Resource-draining chores beyond GPs’ proficiency are dispatched without appropriate instruction or equipment

Horizontal task shifting could sometimes imply more serious medical hazards for the patient subsequent to lack of skills, tools or inadequate organization in general practice. Several comments reported tasks related to follow-up of severe medical conditions without adequate instructions or quality assurance forwarded from the dispatcher. Such situations involved patients with different types of cancer, where the discharge summary from the hospital specialist terminated their relationship with the patient, while the GP was assigned full responsibility for subsequent controls, sometimes for a period of years. Two contributors presented letters from the hospital about patients treated for colon cancer, with specified lists of tests and examinations intended to be conducted going forward, but with no system for consultancy or updating. Another contributor mentioned a patient treated for breast cancer, where the GP was supposed to oversee mammography referral and unspecified clinical examination without further dialogue with the hospital. It was not clear whether all or some tests should be accomplished at every follow-up visit. Moreover, a lack of accountability for keeping an eye on future guideline changes of was emphasized, as guidelines are continuously updated. Another contributor revealed safety hazards:

"I am not sure that we as GPs are sufficiently well prepared for this. Are GPs able to update appropriately regarding follow-up of different types of cancer, and how can this be adequately compatible with patient safety?" (Liza)

Several comments concerned procedures relocated from hospital care to general practice without transferal of required skills, tools or time. One contributor referred to a message from the municipality informing the GP that local dialysis treatment would be offered within the next three weeks. The hospital specialist would oversee the program, but the GPs would have to manage problems on daily basis. Still, the GPs had not been involved in dialogues, training or time planning. Another example dealt with a rheumatologist to whom the GP had referred a patient. The rheumatologist suggested that the Schirmer test and sialometric measurements should first be conducted by the GP. Finally, several contributors described hospital surgeons requesting preoperative assessments from the GPs beyond their level of competency. One such case concerned potential side effects from anticoagulation treatment. One contributor explained why she became provoked:

"The belief in what the GP can achieve, is steadily increasing. Today, I received a letter from a neurologist to whom I had referred a patient. He recommended me to conduct a full metabolic screening of blood, urine and spinal fluid. I am really looking forward to start doing spinal puncture in my office." (Dorothy)

### Potential malpractice is imposed on GPs by hospital colleagues overruling the GPs’ medical judgement

Contributors also presented examples of how task shifting might endanger patients by increasing the risk of missing vital treatments or being exposed to medical malpractice. Many had experienced incidents where hospital colleagues ordered them to start or continue treatments which the GPs knew were not compliant with guidelines or recommended practice. One GP attended a discharge meeting for one of her patients who had been hospitalized for a long time. The patient had admitted side use of illegal drugs but still received strong addictive drugs from the hospital specialist. The GP was requested to continue this prescription, which she found outrageous. Another contributor was told by the hospital that it was acceptable to check urine samples when considering the issuance of a driver’s license for a patient with an established drug addiction without witnessing the sampling, as opposed to guidelines. Other contributors reported that they had been asked to continue prescribing potentially risky medication, such as isotretinoin, which in Norway requires monitoring by a dermatologist. One GP told about being ordered to prescribe strong medication to a patient with addiction problems:

"I really want to fight fire with fire here and tell my patient that I cannot accept such prescriptions (…), but am I entitled to refuse this task when the hospital has ordered me? And what about confronting my patient like this, is it unfair?" (Pat)

Some contributors had experienced incidents where specialists had informally shifted tasks by altering the frames for the GPs’ mandate and function. One example involved patients with potentially serious illnesses not being given priority for access to the hospital. One GP told about a depressed patient who had recently been rescued after an overdose. The GP referred the patient to emergency psychiatric examination, but the clinic told the patient to contact community services. Other challenging situations included the ambulance or the accident & emergency department refusing to accept patients with potentially serious heart disease for hospital assessment. One GP had been told that new guidelines now allowed the emergency services to override the GP’s judgement about whether to bring the patient to hospital or not. The contributor was very upset:

"Who will be responsible if a patient with NSTEMI infarction dies in my office because the ambulance refuses to bring him to the hospital?" (John)

## Discussion

Adverse events associated with horizontal task shifting included spill-over work dispatched to GPs, generating hassles and potentially hazardous delays. Overdiagnosis, reduced access and endangered accountability are associated with time-consuming procedures and required pre-investigations before referral. Chores far beyond GPs’ proficiency and resources are dispatched without adequate support and potential malpractice is imposed on GPs when hospital colleagues overrule the GPs’ medical judgement.

### Strengths and weaknesses

Analysis of data from social media raises several challenges [18]. The actual discussion group was self-recruited but included a substantial majority of Norwegian GPs. Previous discussions have demonstrated a broad range of attitudes among contributors, more often presenting constructive dialogues than grumbling complaints. Furthermore, the benefit of a forum where such issues may be discussed freely among colleagues, without scaring patients or alarming authorities, had often been demonstrated. Some of the posts we have included, combine letting off steam with providing information relevant for our aim. Contributors did not present extreme positions regarding demography or temperature of dialogue and we assess external validity as satisfactory. Most of the texts presented surprisingly thick descriptions of the actual events [14], even the short ones. We suggest that our interpretations and findings are transferable to health care systems with the GP in a gatekeeper function comparable to Norway [19]. In other settings, our findings could be relevant and transferable by encouraging an awareness of factors essential for appropriate task shifting planning.

The contributions were retrospectively enrolled and were not responses to pre-established research questions or triggers. A few contributors applied more specific terms, like task shifting or patient safety, but most texts were written in everyday language. To sustain internal validity, we made efforts to not overinterpret the meaning of the content, although we aimed for synthesis of the contributions. Furthermore, we explored experiences shaped by impressions and emotions of the involved contributors—subjective phenomena supporting the internal validity of the phenomenon under study. We also acknowledge that being GPs ourselves, we know and care more about the primary care side of these interaction than of the counterparts’ perspectives.

Braithwaite argues that since different stakeholders have distinguishable views on what is happening, tailoring change to the circumstances is crucial [20]. Recognizing these situations from our own practice experiences have probably helped us interpret the context and meaning. However, they have also potentially obstructed our understanding of the situation facing the colleagues from whom tasks were transferred.

### What is known from before? What does this study add?

Substantial evidence exists about the impact of vertical task shifting on outcomes such as cost savings, efficiency improvements, quality of care, user satisfaction or health equity related to conditions such as infectious diseases [2,21], mental health problems [22] or childbirth [23]. Evidence is mostly developed from low- and middle-income countries, but it also refers to high-income settings [24]. However, our priority was to study patient safety consequences regarding horizontal shifting of tasks from specialist colleagues and other service partners to GPs, a strategy identified as a critical element of the GP crisis in Norway [25]. The consequences of vertical task shifting currently carried out from GPs to midwives and nurses, such as Pap smears, contraception and follow-up of patients with chronic diseases, are vividly discussed among GPs in Norway. However, this was not our focus in this study.

To our knowledge, horizontal task shifting has been substantially less studied. Recent discussions among GPs in Norway have predominantly dealt with the negative effects of horizontal task shifting on workload, which may also represent a threat to patient safety. Specifically, our study adds to knowledge about the potential impact of such task shifting on different adverse events. Initially, we thought that informal task shifting with lower levels of agreement on guidelines and procedures would raise more challenges for patient safety than formal task shifting. Yet, in studying the contributions, we realized that formal task shift based on e.g. national guidelines was no guarantee for patient safety. Although we emphasized accounts about informal cases, we also included some adverse events related to formally staged horizontal task shifting. These accounts describe how national guidelines, developed with minimal GP impact, have been implemented and transformed.

Our data do not present conclusions about the factual consequences of the specific reported incidents. Such information would have extended the perspectives of our study but was not available. Still, events are conceptually defined as adverse if they represent potential harm of patient safety [12], thereby deserving attention as warning lights for quality improvement on a system level. Hence, how can our findings be transformed to preventive action and quality improvement?

### Strategies and measures to prevent adverse events

Contributors presented a wide range of incidents, from minor hassle for patient and doctor to potentially serious adverse events (delay of cancer diagnosis, insufficient treatment due to lack of resources, or risk of malpractice). Our analysis indicates that individual-, organizational- and system-level mechanisms interact when horizonal task shifting endangers patient safety. The problems will be solved neither by criticizing colleagues for unilateral workload pushing nor by dismissing horizontal task shifting to GPs in general [4,20,26].

Firstly, attention must be drawn towards the complex system-level mechanisms responsible for creating and maintaining this stream of hazards. Development and implementation of guidelines and strategies implying horizontal shifting of tasks should always be scrutinized regarding risk and vulnerability and developed in negotiations and collaboration between all participating stakeholders. Better overview and planning of resources such as competence, equipment and information must be addressed. Such measures could help counteract overdiagnosis propensities and their consequences [27], such as reduced access and endangered accountability.

Secondly, involving all relevant stakeholders is an essential pathway to prevent patient safety hazards [20,26]. Our findings should lead to increased attention among managers of hospital services and improvement teams towards consequences of high workload and time pressure in their organizations, combined with ideas following the coordination reform that GPs are the real coordinators of patients’ health care [8]. Our analysis demonstrated how such attitudes may unintentionally lead to spill-over work from hospital colleagues to GPs. Nancarrow [4] discusses different ‘demarcationary strategies’ for creation and control of inter-professional occupational boundaries, respectively understood as a consensual shift in boundaries, based on mutual negotiation or the competitive, conflictual processes of occupational imperialism. Some of our contributors reported positive experiences regarding direct responses to the colleague concerning unfounded horizontal task shifting. Easily accessible systems for direct communication between first- and secondary line specialists as suggested by the Norwegian College of General Practitioners could be a low-threshold tool for adjusting task shifts in appropriate directions [28]. Following Denmark, Norway formally established the Practice Consultant Organization in 1995 for collaboration between primary and secondary health care, with GPs employed as intermediaries (PCs) by hospital departments [29]. The PCs aim to improve procedures and communication on a system level and are not supposed to resolve individual cases. While this system represents a key potential for local improvement, our analysis indicates that some of the reported recurrent problems require national policy strategies specifically devoted to the impact of horizontal task shifting.

Finally, GPs are not negative to task shifting itself, neither vertical nor horizontal, when purposes and frameworks are adequately negotiated and handled. Guidelines presenting advice and examples of task shifting elaborated as for example shared care with medication and prescription represent concrete and specific efforts to develop procedures intended to prevent adverse effects of collaboration across health care system levels [30]. These guidelines do not, however, present convincing evidence of successful functioning. In this regard, they share the best intentions underlying some of the task shifts we have presented as less efficacious regarding patient safety. Clinical pathways for patients with serious or chronic diseases could benefit from the continuity of care in general practice combined with thoroughly planned formal horizontal task shifting with appropriate supply flow [31–33]. Under such circumstances, the GP—supported, but not commanded by hospital specialist colleagues—may offer unique long-term care for patients with conditions such as cancer, chronic respiratory or cardiac disease or progressive neurological diseases. Essential for such care pathways is a mutually respectful and collaborative environment where primary and secondary health care support each other. This would ensure that the patients are taken care of by a safe, complete and coordinated system. However, under the current circumstances, proactive planning of such task shifting is not easy.

## Implications

Patient safety is endangered when horizontal task shifting is initiated and performed without a systematic process considering available resources and involving all stakeholders. Development and implementation of guidelines and strategies for task shifting require that risk and vulnerability analysis be scrutinized to secure competence, resources, time and equipment before launching. Infrastructures comprised of local coordination groups may facilitate dialogue between health care service levels and negotiate responsibilities and workload.
